# Closing Europe’s Cardiovascular Implementation Gap: A Critical Review of the Safe Hearts Plan and the Cyprus Test Case

**DOI:** 10.3390/jcm15176614

**Published:** 2026-08-27

**Authors:** Georgios P. Georghiou, Sotiris Kyriakou, Panos Georghiou, Amalia Georgiou, Nikolas Iosif, Konstantinos Toutouzas, Andrew Xanthopoulos, Konstantinos Lampropoulos, Argyris Kyriakou, Filippos Triposkiadis

**Affiliations:** 1 Department of Surgery, Gray Faculty of Medical & Health Sciences, Tel Aviv University, Tel Aviv 6997801, Israel; 2 School of Medicine, European University Cyprus, Nicosia 2404, Cyprus; ktoutouz@gmail.com (K.T.); andrewvxanth@gmail.com (A.X.); k.lampropoulos@euc.ac.cy (K.L.); ftriposkiadis@gmail.com (F.T.); 3 Barts and The London School of Medicine and Dentistry, Queen Mary University of London (QMUL), London E1 4NS, UK; s.kyriakou@nhs.net (S.K.); panos.georghiou@gmail.com (P.G.); 4 Krankenhaus Maria-Hilf, 47805 Krefeld, Germany; amalia.georgiou.med@gmail.com; 5 Klinik für Gastroenterologie, Hepatologie, Endokrinologie, Diabetologie, Helios Universitätsklinikum Wuppertal, 42283 Wuppertal, Germany; nikolas.iosif.md@gmail.com; 6 First Department of Cardiology, Medical School, National and Kapodistrian University of Athens, 11527 Athens, Greece; 7 Department of Cardiology, Faculty of Medicine, University of Thessaly, 41110 Larissa, Greece; 8 Barking, Havering and Redbridge University Hospitals NHS Trust, London RM7 0AG, UK; argyris.kyriakou6@nhs.net

**Keywords:** cardiovascular disease, European Safe Hearts Plan, implementation science, acute coronary syndrome, heart failure, atrial fibrillation, digital health, Cyprus

## Abstract

Cardiovascular disease remains Europe’s leading cause of death and disability despite mature evidence for prevention, acute and chronic treatment, rehabilitation, and secondary prevention. This structured critical narrative review evaluates whether the European Safe Hearts Plan can convert this evidence into coordinated, measurable, and accountable cardiovascular pathways. We appraise the plan’s ten flagship actions by evidence maturity and implementation readiness; distinguish shared system functions from disease-specific delivery for acute coronary syndromes, heart failure, and atrial fibrillation; compare selected European Society of Cardiology and American Heart Association/American College of Cardiology recommendations; and propose a core indicator set and staged Cyprus pilot. Guideline-supported interventions should be standardised and audited, whereas screening technologies, digital health, artificial intelligence, and novel phenotyping require governed implementation and prospective assessment of patient outcomes, costs, equity, and unintended consequences. Cyprus is presented as a bounded implementation setting rather than a directly generalisable European model. The plan should be judged through a limited set of clinical, implementation, and equity indicators, including premature cardiovascular mortality, risk factor control, guideline-directed therapy, rehabilitation participation, registry completeness, reach, and fidelity.

## 1. Introduction

Cardiovascular disease (CVD) remains Europe’s largest cause of mortality and one of its greatest sources of disability, hospital use, productivity loss, and informal care dependency. Recent European and global estimates put cardiovascular mortality at roughly 1.7 million deaths each year in the European Union and close to 3.9 million across the wider European region. This burden persists despite decades of progress in acute coronary care, stroke care, lipid lowering, antihypertensive treatment, and smoking reduction. Demographic ageing and the rising prevalence of obesity, diabetes mellitus, chronic kidney disease, atrial fibrillation, and multimorbidity mean that the absolute number of people living with CVD is likely to remain high, even where age-standardised mortality continues to decline [1,2,3,4,5,6].

The burden is distributed unevenly across Europe, with worse outcomes in settings where prevention infrastructure is weaker, socioeconomic resources are fewer, specialist access is poorer, and guideline-directed therapy is implemented less consistently. This pattern exposes a delivery problem in which avoidable cardiovascular harm increasingly depends on whether effective care can be organised, financed, measured, and sustained across the life course [1,2,7,8,9].

The economic consequences are equally important because cardiovascular disease remains one of Europe’s most expensive disease groupings. The annual burden in the European Union was estimated at €282 billion in 2021 after direct medical costs, lost productivity, and informal care were included. Prevention therefore functions as a health system sustainability strategy as well as a public health priority, since inadequate early detection, rehabilitation, and long-term follow-up continue to convert preventable risk into acute events and chronic disability [4,6,10,11].

Europe’s problem is not a shortage of cardiovascular evidence but the uneven way this evidence is organised and delivered. The continent has strong guidelines, mature scientific evidence, and several national initiatives, yet many patient pathways still pass through inconsistent prevention, heterogeneous screening, underused rehabilitation, fragmented digital systems, and unequal access to specialist care. This fragmentation creates preventable delays and recurrence while reinforcing inequalities between and within countries [12,13,14,15,16].

The central translational question is whether Europe can turn established cardiovascular knowledge into a joined-up model of care that clinicians can use, health systems can measure, and patients can hold accountable. The European Safe Hearts Plan is important because it provides a framework through which Europe’s prevention delivery gap can be examined across prevention, detection, treatment, rehabilitation, data, digital infrastructure, research, financing, and equity [17,18]. Its value would depend on whether it can move cardiovascular prevention from a collection of guidance documents and local initiatives into a coordinated programme with defined responsibilities, common indicators, sustainable funding, and transparent accountability. Cyprus and its General Healthcare System are considered here one bounded environment in which this model could be designed, measured, and refined through implementation.

**Figure 1:**
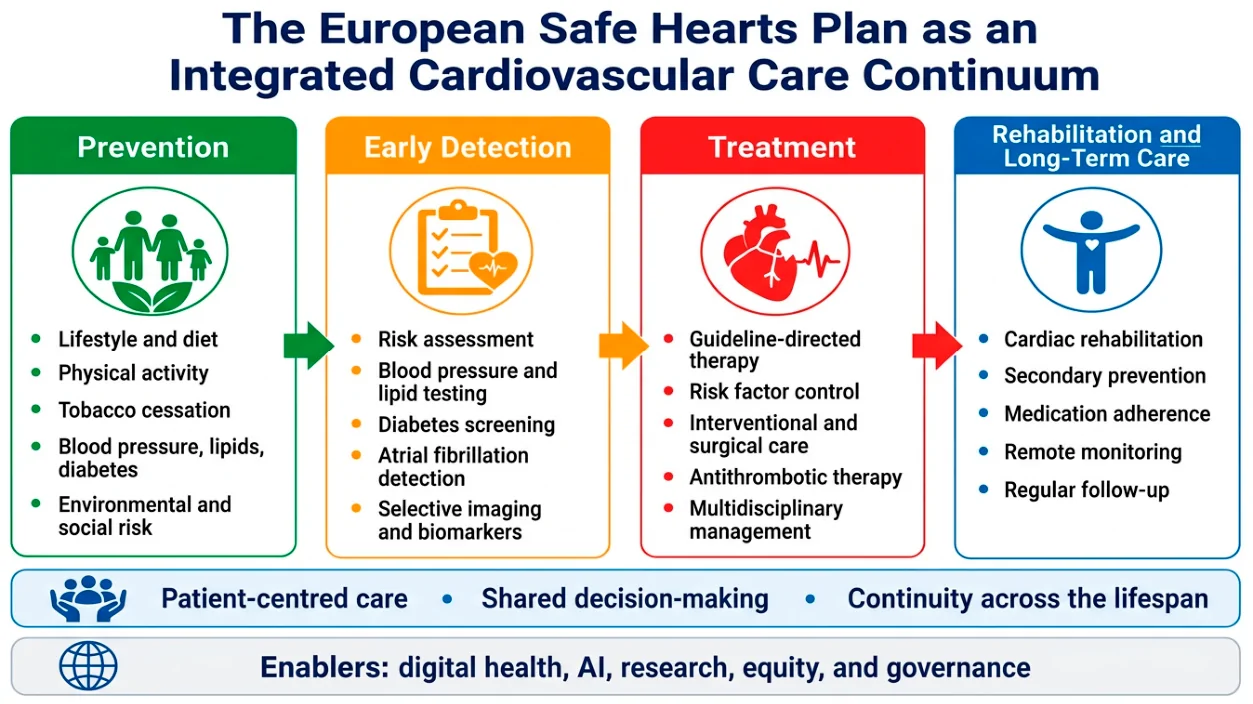
The European Safe Hearts Plan as an integrated cardiovascular care continuum. This figure presents the Safe Hearts Plan as a life course cardiovascular pathway linking prevention, early detection, treatment, rehabilitation, and long-term care. Prevention includes lifestyle, physical activity, tobacco cessation, cardiometabolic risk factor control, and environmental and social risk reduction. Early detection includes risk assessment, blood pressure and lipid testing, diabetes screening, atrial fibrillation detection, and selective imaging or biomarker use. Treatment includes guideline-directed therapy, risk factor control, interventional and surgical care, antithrombotic therapy, and multidisciplinary management. Rehabilitation and long-term care include cardiac rehabilitation, secondary prevention, medication adherence, remote monitoring, and regular follow-up. Cross-cutting principles include patient-centred care, shared decision-making, continuity across the lifespan, digital health, artificial intelligence, research, equity, and governance. Abbreviation: AI, artificial intelligence.

The purpose of this review is therefore deliberately narrower than the development of another clinical guideline. It asks four implementation questions: which Safe Hearts components are supported by mature clinical or public health evidence; which remain implementation hypotheses or emerging technologies; how shared system infrastructure should be translated into disease-specific pathways; and which indicators and decision gates should determine continuation, adaptation, or discontinuation. This review contributes four practical outputs: an evidence readiness map of the ten flagship actions, a staged Cyprus implementation roadmap, an ESC–AHA/ACC pathway crosswalk, and a core performance scorecard. Figure 1 locates these outputs within a continuous cardiovascular pathway.

An earlier narrative review from our group set out the policy case for an EU cardiovascular action plan [19]. The present review moves beyond that advocacy by critically appraising the ten now-defined Safe Hearts flagship actions according to evidence maturity and implementation readiness; translating shared infrastructure into disease-specific ACS, HF, and AF pathways; and specifying a core KPI scorecard and staged Cyprus decision gates. Its incremental contribution is therefore an operational and evaluative architecture, rather than another general call for European action.

## 2. Review Approach and Analytical Outputs

This manuscript is a structured critical narrative synthesis of evidence relevant to European cardiovascular prevention, delivery, and implementation. Priority was given to official and primary sources, including European Society of Cardiology guidelines and scientific statements, World Health Organization and European Commission policy documents, European epidemiological and economic datasets, major prevention and screening studies, Cyprus-specific sources, peer-reviewed environmental and vaccination evidence, and implementation science frameworks. Evidence was interpreted by maturity, implementability, and relevance to cardiovascular pathway design, with institutional items retained where they remained the most appropriate source for the policy or programme being cited.

Sources were selected purposively rather than through a systematic review search protocol. Inclusion was based on direct relevance to the plan’s policy functions, disease pathways, implementation determinants, or measurement framework. Preference was given to current ESC guidelines and official European policy sources; major trials, economic evaluations, registries, and implementation frameworks were used where they tested feasibility, outcomes, costs, or transferability. AHA/ACC guidance was included as an external comparator for selected pathways, not as an exhaustive transatlantic guideline review. This approach improves traceability while preserving this manuscript’s intended critical narrative design.

Evidence was weighted qualitatively through a set of linked appraisal questions that preserve the critical review character of this manuscript. We considered whether the source base had reached guideline-level maturity; whether outcomes were patient-centred or limited to detection, process, or surrogate measures; whether major limitations such as selection bias, false-positive burden, implementation cost, and external validity were explicit; whether delivery required new digital or workforce capacity; and whether transferability had been tested outside selected settings. This weighting is not a formal grading system, since this review is neither a guideline nor a systematic review, but its purpose is to separate established clinical evidence from implementation-dependent propositions.

For each proposed component, implementation readiness was interpreted separately from evidence maturity. ‘Ready for standardisation’ denotes an intervention supported by mature recommendations and a feasible delivery pathway; ‘ready with adaptation’ denotes an evidence-supported intervention whose organisation depends materially on local context; and ‘pilot before scale’ denotes an approach for which diagnostic performance or policy rationale exceeds evidence for net patient benefit, cost-effectiveness, or sustainable delivery. These categories are interpretative, not formal grades of recommendation.

## 3. Critical Appraisal of Safe Hearts Plan’s Ten Flagship Actions

The Safe Hearts Plan organises European action around prevention, early detection and screening, and treatment and care, including rehabilitation [17]. Its ten flagship actions span public communication, food and tobacco policy, primary prevention, vaccination, cardiovascular health checks, personalised treatment and monitoring, artificial intelligence and digital innovation, inequality monitoring, and a research and innovation roadmap. This breadth is politically useful, but the actions do not share the same evidentiary status or implementation risk. Table 1 therefore links each flagship to its predominant evidence base, readiness category, and the question that should be answered before expansion.

Europe already has several strong cardiovascular policy components across clinical guidance, public health, and European policy. The European Society of Cardiology (ESC) provides detailed guidance for prevention, dyslipidaemia, hypertension, chronic coronary syndromes, and atrial fibrillation [12,13,14,15,16]. The World Health Organization has long promoted non-communicable disease control through population health measures, primary care strengthening, and essential package approaches [21,22]. The European Commission’s Healthier Together initiative provides a cross-disease framework for non-communicable disease action [23], while Europe’s Beating Cancer Plan demonstrates the effect of giving a disease area visible, funded, transnational political priority [24].

These frameworks are scientifically strong, although their combined effect across the cardiovascular continuum remains incomplete. Guidelines define clinical standards once a patient is identified [12,13,14,15,16], public health frameworks describe broad prevention goals [21,22], and European Union initiatives support coordination [23,24]. The missing element is an operational model that specifies how prevention, screening, diagnosis, treatment, rehabilitation, follow-up, data capture, financing, and accountability should interact at the continental scale [17,18].

The added value of the Safe Hearts Plan, if realised, would be to connect ESC guidelines and existing public health frameworks into one cardiovascular pathway. Such a pathway would need clearer responsibility for inequalities, interoperability, financing, implementation, and accountability. Its relevance therefore depends on whether it can organise mature evidence into a measurable programme with national ownership, common indicators, and credible mechanisms for funding and audit.

The comparison with cancer policy is useful when framed with appropriate caution, because cancer and cardiovascular disease differ in biology, public perception, screening paradigms, treatment pathways, and survivorship models. Europe’s Beating Cancer Plan nevertheless shows that a disease area can gain political traction when prevention, early detection, treatment quality, research, data, funding, and equity are organised within one visible framework. Cardiovascular disease requires comparable political visibility, adapted to chronic risk factor control, acute event prevention, rehabilitation, and lifelong follow-up [17,18,24].

The appraisal identifies a central asymmetry. Tobacco control and the systematic treatment of established risk factors are supported by mature evidence but often lack consistent delivery; AI-enabled detection and some population screening approaches may be technically feasible but remain uncertain in terms of net benefit, workflow burden, and affordability. A credible implementation programme should therefore resist uniform roll-out: the former group requires standards and audit, whereas the latter requires prospective pilots with stopping rules.

## 4. Translating Evidence into a Continuous Cardiovascular Care Model

**Prevention.** A credible European cardiovascular strategy should begin before clinical disease appears. The INTERHEART paradigm, later prevention frameworks, and life course constructs converge on the same central lesson: many cardiovascular events arise from modifiable risk exposures that begin early, accumulate over decades, and interact with social context. Prevention therefore requires organised action across the life course and across sectors, since opportunistic advice during specialist encounters cannot carry the burden of population-level risk reduction [25,26,27,28,29].

Hypertension and dyslipidaemia show why implementation matters as much as evidence [13,14,30,31,32,33,34,35]. Both conditions are common, measurable, treatable, and strongly linked to subsequent cardiovascular events. Persistent gaps in control usually arise from incomplete identification, therapeutic inertia, poor adherence, and insufficient follow-up. A European cardiovascular strategy should therefore specify the minimum standards for measurement, treatment intensification, follow-up intervals, and performance reporting.

Cardiometabolic prevention must also address diabetes and obesity explicitly. The expanding diabetes burden and current diabetes–cardiovascular guidance support systematic glycaemic assessment and multifactorial risk reduction [36,37,38]. Obesity should be treated as a chronic, relapsing disease; interventions may include structured behavioural treatment and, for eligible adults, evidence-based pharmacotherapy that can produce substantial weight loss, with safety, affordability, persistence, and equitable access monitored [39,40,41].

Life course prevention also needs to extend beyond the first clinical event. In real health systems, prevention begins with primordial and primary prevention, continues through secondary prevention, and then becomes long-term risk control after established disease. The Safe Hearts framework should therefore connect prevention and rehabilitation structurally, because separating them into different silos undermines continuity [26,27,28,29,42,43,44].

**Risk-based detection.** Early detection forms the second policy pillar, although the case for screening must remain disciplined and outcome-facing. Earlier diagnosis creates value only when the target population is appropriate, the method is reliable, the downstream pathway can deliver treatment or follow-up, and the health system can monitor unintended consequences such as false positives, anxiety, and inequitable uptake [45,46,47]. The defensible position is therefore risk-based and evidence-based screening, with safeguards against indiscriminate testing [48,49,50,51].

Atrial fibrillation illustrates the promise and the caution required in screening policy. Opportunistic and technology-enabled detection can identify previously unrecognised atrial fibrillation, although trial results differ according to age, device, and strategy, and higher detection does not always translate directly into improved hard outcomes. Screening should therefore focus on older or higher-risk populations, with confirmatory pathways and anticoagulation decision-making in place before technology is scaled [16,48,49,50,51].

Major screening studies illustrate why evidence weighting is necessary as systematic atrial fibrillation screening can identify previously undiagnosed disease and may produce modest net clinical benefit in selected older populations. However, implantable loop recorder strategies increased atrial fibrillation detection and anticoagulation without showing a clear proportional reduction in stroke outcomes. Large-scale smartwatch and photoplethysmography studies show feasibility and patient reach, although they also highlight selection, confirmatory testing, false-positive, and downstream management limitations [48,49,50,51].

**Treatment, rehabilitation, and follow-up.** A European cardiovascular strategy should extend beyond prevention and diagnosis. Treatment quality still varies across Europe, and rehabilitation remains underused despite its contribution to functional recovery, risk factor control, recurrent event reduction, and quality of life. When rehabilitation is treated as an optional post-discharge service, cardiovascular care becomes episodic and incomplete [15,16,42,43,44].

Cardiac rehabilitation should be positioned as an essential cardiovascular service, with an evidence base supporting exercise-based, home-based, and structured secondary prevention models and with telerehabilitation extending access where geography, workforce capacity, or patient preference limits participation in centre-based programmes [42,43,44,52].

**Digital infrastructure and accountability.** Digital health and artificial intelligence are relevant to the Safe Hearts Plan when they support continuity, access, and measurement rather than add another layer of complexity. Digital health earns its place by closing gaps in follow-up, interoperability, and patient engagement [53,54,55,56]. Artificial intelligence should be judged by whether validated tools improve decisions, reduce inequity, fit clinical workflow, and operate within acceptable standards of governance, explainability, privacy, and interoperability [57,58,59,60].

Remote monitoring, telemedicine, wearable technologies, interoperable electronic health records, registry-linked analytics, and digital rehabilitation can strengthen the cardiovascular continuum when they are designed around clinical pathways. These tools are especially valuable where geography, workforce shortages, or follow-up attrition undermine continuity. The priority should be interoperable digital infrastructure that follows the patient across prevention, primary care, acute care, rehabilitation, and long-term management [52,53,54,55,56,61,62].

Registries are the practical link between policy ambition and accountability. Without interoperable registries, European cardiovascular policy remains dependent on episodic audits, local enthusiasm, and incomplete data. The EuroHeart and ESC Atlas models show how structured data systems can support benchmarking, quality improvement, and cross-country learning. An implementation programme would need a minimum dataset, common definitions, and the routine public reporting of process, outcome, and equity indicators [61,62].

**Equity and environmental determinants.** A European cardiovascular strategy cannot treat inequality as an afterthought, because socioeconomic deprivation, education, geography, social exclusion, and environmental exposure shape risk factor prevalence, diagnostic delay, treatment access, rehabilitation participation, and long-term outcomes, influencing both how risk is generated and how care is received [7,8,9].

Air pollution and climate-related exposure extend the cardiovascular agenda beyond the clinic, affecting cardiovascular events and vulnerability directly, so that prevention cannot be framed only as individual behaviour change but must connect cardiovascular health with public health regulation, urban design, air quality policy, climate adaptation, and heat risk planning [63,64,65,66,67].

This issue is particularly important for digital health, as technologies that depend on smartphones, continuous connectivity, health literacy, and active patient engagement can widen disparities if safeguards are absent [7,8,9]. Digital inclusion, language access, clinician support, community outreach, and alternative non-digital pathways should be built into implementation design from the start [53,54,55,56].

These elements become clinically meaningful only when linked by explicit hand-offs. A primary care risk assessment must trigger treatment and recall; an abnormal rhythm signal must trigger confirmatory electrocardiography and an anticoagulation decision; an acute admission must trigger discharge therapy, rehabilitation referral, and early follow-up; and every transition must populate a common minimum dataset. The shared model is therefore infrastructure for disease-specific care, not a substitute for it.

## 5. Cyprus as a Bounded Implementation Pilot

Cyprus is proposed as a bounded pilot because its national scale, universal General Healthcare System, developing digital infrastructure, and identifiable academic–clinical networks make the complete policy-to-outcome chain comparatively observable [68,69,70]. The claim is methodological rather than representative: Cyprus can test whether an integrated pathway is feasible, adopted, affordable, equitable, and sustainable but cannot by itself establish direct applicability to larger European systems.

Cyprus is included for methodological as well as policy reasons. Implementation science often needs bounded settings in which the full pathway can be observed from policy decision to primary care identification, specialist referral, rehabilitation, digital follow-up, registry reporting, financing, stakeholder response, and equity monitoring. Cyprus offers that tractability, and its value would lie in identifying which functions of an integrated cardiovascular strategy are essential, measurable, affordable, and transferable across more complex European systems.

Nevertheless, caution is required when extrapolating the findings from Cyprus to larger and more heterogeneous European healthcare systems. European countries differ substantially in population demographics, cardiovascular burden, healthcare financing, workforce capacity, governance structures, digital maturity, rehabilitation infrastructure, and resource availability. Implementation strategies that prove feasible in Cyprus may therefore require substantial contextual adaptation before being transferred elsewhere. The Cyprus model should be regarded as an implementation laboratory capable of generating real-world evidence on feasibility, acceptability, fidelity, clinical effectiveness, cost-effectiveness, equity, sustainability, and operational barriers, rather than as a directly generalisable template [68,69,70,71,72,73].

**Figure 2:**
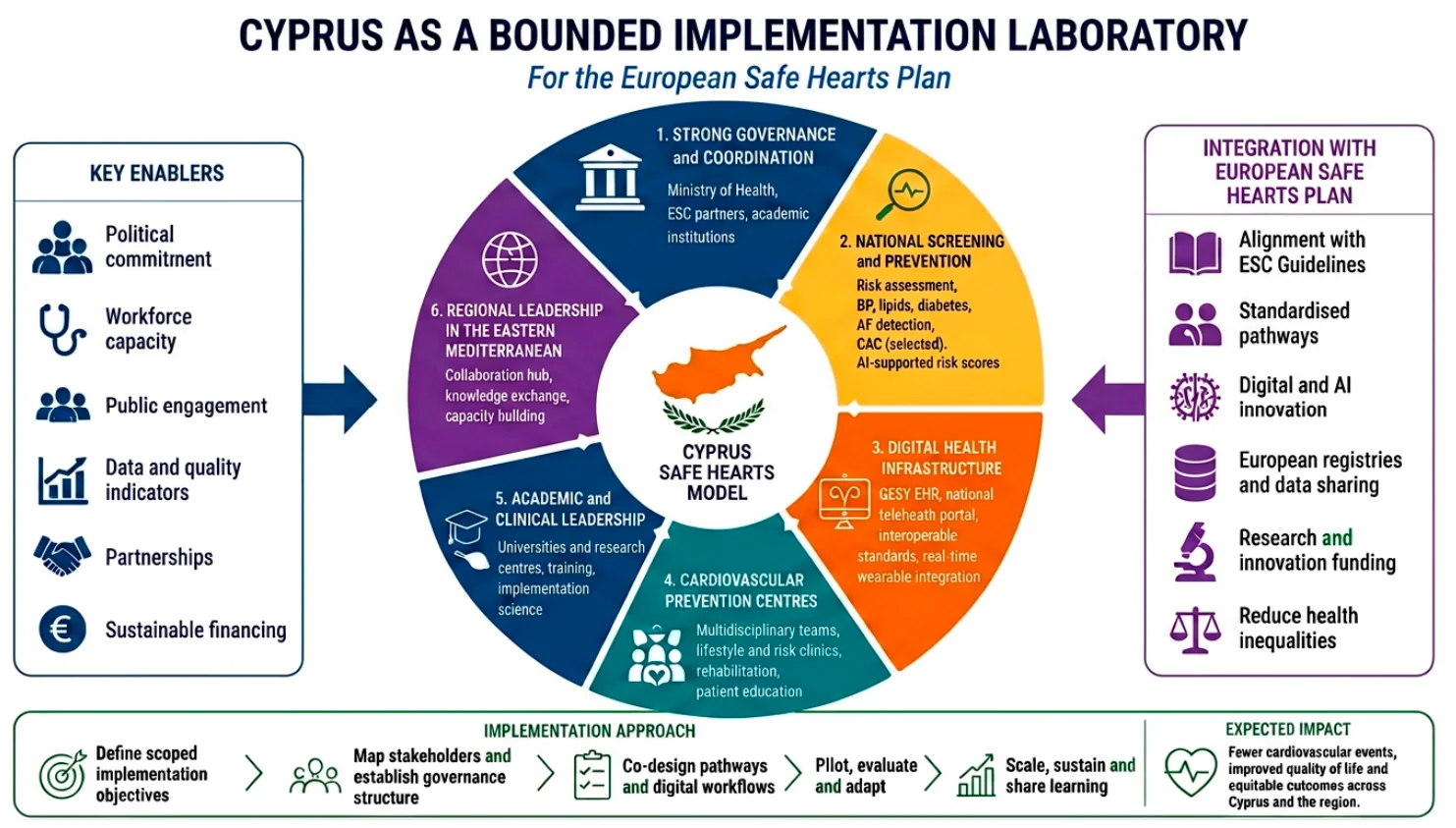
Cyprus as a bounded implementation laboratory for the European Safe Hearts Plan. Cyprus is presented as a testable national platform rather than as a directly generalisable European model. Universal coverage through the General Healthcare System, manageable population scale, bounded geography, developing digital infrastructure, and academic–clinical links could allow pathway feasibility, stakeholder adoption, costs, equity effects, and transferability conditions to be evaluated before wider European adaptation. Abbreviations: AF, atrial fibrillation; AI, artificial intelligence; CAC, coronary artery calcium; EHR, electronic health record; ESC, European Society of Cardiology; GESY, General Healthcare System.

These features make Cyprus a practical place to test whether an integrated cardiovascular strategy can be delivered under real health system conditions. A national Safe Hearts pilot could link structured primary care risk assessment with referral into multidisciplinary Cardiovascular Prevention Centres, integrated digital follow-up, national rehabilitation standards, registries aligned with EuroHeart principles, and mandatory indicators reported at fixed intervals [61,62,68,69,70]. Academic institutions could then evaluate implementation through established frameworks that examine adoption, reach, fidelity, feasibility, sustainability, cost, and equity [71,72,73]. Figure 2 illustrates how Cyprus could function as a bounded implementation laboratory for evaluating governance, prevention pathways, digital infrastructure, academic–clinical collaboration, equity, cost, adoption, and transferability before broader European adaptation.

A credible Cyprus roadmap would need staged implementation with predefined decision points. The design phase would establish governance, define the minimum dataset, agree on referral criteria, specify outcome measures, and secure financing [68,69,70,71,72,73]. Deployment would introduce screening, prevention centre referral, rehabilitation standards, and digital follow-up across selected districts or provider networks. Scale-up would extend successful components nationally only if transparent dashboards show acceptable uptake, quality, outcomes, cost, and equity [71,72,73,74,75].

The proposed phases, indicative timelines, core actions, and decision gates are summarized in Table 2.

External validity should be assessed through explicit boundary conditions: healthcare financing and purchaser incentives; primary care and specialist workforce; emergency and referral organisation; digital maturity and interoperability; registry completeness; rehabilitation capacity; governance centralisation; public trust; and baseline social and geographic inequalities. Larger, decentralised, or insurance-fragmented systems may reproduce the pilot’s functions but not its organisational form. Transferability should therefore be demonstrated by adaptation studies in contrasting settings, with the reporting of implementation friction, resource use, fidelity, and negative results [71,72,73].

## 6. Disease-Specific Pathways, Guideline Comparison, and Remaining Evidence Gaps

A general cardiovascular framework needs disease-specific operating pathways. Because the intended setting is Europe, ESC recommendations remain the primary clinical standard; selected AHA/ACC documents are used to identify convergent delivery functions and material differences requiring local adaptation. The comparison is deliberately implementation-focused and does not replace full guideline appraisal. Risk equations, treatment thresholds, regulatory environments, and service configurations are not automatically interchangeable across continents.

The disease-specific guideline anchors, minimum delivery bundles, and illustrative pathway indicators are summarized in Table 3.

**Prevention pathway.** The operational sequence is measurement, validated regional risk estimation, treatment, and recall. SCORE2/SCORE2-OP support European risk estimation, whereas US guidance uses different equations and treatment conventions [12,27,45,46,47,76]. A material implementation difference is blood pressure classification and targeting: ESC guidance retains hypertension at ≥140/90 mmHg and targets a treated systolic pressure of 120–129 mmHg when tolerated, whereas US guidance labels 130–139 or 80–89 mmHg as stage 1 hypertension and uses a general target below 130/80 mmHg [14,76]. A Safe Hearts pathway should therefore document the selected risk tool, diagnostic definitions, treatment thresholds, intensification rules, and subsequent risk factor control rather than importing them uncritically.

**ACS pathway.** Implementation begins before hospital arrival and continues beyond discharge. The European and US guidance converge on coordinated emergency networks, prompt diagnosis and reperfusion or invasive assessment, evidence-based antithrombotic and lipid-lowering therapy, and secondary prevention [77,78]. The implementation unit should therefore be the entire EMS–hospital–rehabilitation–follow-up pathway, with time metrics, contraindication-adjusted discharge therapy, rehabilitation participation, and recurrent events measured together.

**HF pathway.** A Safe Hearts HF pathway should secure timely diagnostic testing, define phenotype, initiate indicated therapy early, titrate after discharge, and coordinate congestion, renal function, iron status, devices, rehabilitation, and advanced or palliative care [79,80,81]. The 2023 ESC focused update gives SGLT2 inhibitors a Class I, level A recommendation in HFmrEF and HFpEF, whereas the 2022 AHA/ACC/HFSA guideline assigns a Class IIa recommendation in those phenotypes [79,80,81]; this difference partly reflects guideline timing and should be revisited when guidance is updated. Reporting only prescriptions at discharge is insufficient: medication eligibility, contraindications, tolerated dose, early follow-up, readmissions, quality of life, and persistence should be captured.

**AF pathway.** Both ESC and US guidance require ECG-confirmed diagnosis, the prevention of thromboembolism, risk factor and comorbidity management, symptom-directed rate or rhythm control, and reassessment [16,82]. Their anticoagulation frameworks are not identical: the 2024 ESC guideline uses CHA2DS2-VA, recommends anticoagulation at a score ≥ 2, and advises considering it at a score of 1; the 2023 US guideline instead centres treatment on an estimated annual thromboembolic risk ≥ 2%, supported by a validated score such as CHA2DS2-VASc [16,82]. Screening therefore has value only if a positive signal reaches a jurisdiction-specific pathway. A registry should distinguish device alerts from confirmed AF and should measure anticoagulation appropriateness, stroke, major bleeding, symptoms, and access to rhythm control options.

**Screening benefit, harm, and cost.** Screening may generate overdiagnosis, false-positive alerts, anxiety, incidental findings, confirmatory testing, specialist referrals, and treatment of uncertain net value. These downstream effects consume staff and diagnostic capacity and can displace higher-value care. Evaluations should therefore prespecify patient-important outcomes, adverse effects, quality-adjusted life-years, budget impact, and distributional effects—not diagnostic yield alone. The STROKESTOP economic analysis used a Markov cohort model informed by a median 6.9-year follow-up of intermittent ECG screening and extrapolated outcomes over the remaining lifetime; it estimated screening to be cost-saving in the selected Swedish cohort. In contrast, the LOOP evaluation was a prespecified three-year within-trial cost–utility analysis of implantable loop recorder screening and found higher costs, minimal QALY gain, and a low probability of cost-effectiveness [48,49,83,84]. These estimates are not directly discordant: they reflect different populations, monitoring modalities, intervention costs, time horizons, and modelling assumptions. They support strategy-specific prospective economic evaluation rather than a generic endorsement or rejection of AF screening.

**Modified Haller index as an exploratory AF phenotype.** Thoracic morphology has been proposed as a complementary marker in AF. In the EORP-AF registry, asymptomatic presentation was common and associated with poorer one-year outcomes, although that study did not assess thoracic shape [85]. A separate retrospective study of 115 patients undergoing electrical cardioversion for persistent AF reported a lower modified Haller index (MHI) in asymptomatic patients and proposed a threshold below 2.2 [86]. The MHI is radiation-free but combines an external latero-lateral thoracic measurement with an echocardiographic anteroposterior diameter; it is not yet a stand-alone bedside screening test. The evidence is hypothesis-generating: incremental discrimination beyond validated clinical variables, reproducibility, performance in unselected populations, effects on management or outcomes, and cost-effectiveness remain unproven. It should therefore be studied prospectively within governed phenotyping research, not incorporated into routine population screening at present.

**Digital and AI lifecycle.** Real-world deployment requires more than validation at release. Systems must address algorithmic bias, calibration drift, interoperability with heterogeneous records, cybersecurity and privacy, regulatory classification, procurement and reimbursement, clinician acceptance, alert fatigue, patient access, vendor dependence, maintenance, version control, incident reporting, and safe decommissioning. The EU Artificial Intelligence Act and European Health Data Space create relevant regulatory and data governance obligations [87,88]. Each AI-supported decision system should therefore have an accountable owner, intended use statement, local validation, human oversight protocol, equity audit, update plan, recurring performance review, and a predefined suspension threshold.

## 7. Core Implementation Indicators and Priority Recommendations

A compact scorecard is preferable to a long catalogue that cannot be audited. The core set in Table 4 combines outcomes, delivery, data quality, implementation, and equity. Definitions, denominators, exclusions, reporting intervals, and stratifiers should be harmonised before comparison; countries may add local measures but should not replace the common core. Targets should be time-bound and calibrated to baseline performance rather than selected after implementation.

Based on this evidence, the Safe Hearts Plan should be implemented through seven priority actions:

1. Require each participating country to publish a funded national cardiovascular plan with named accountable leadership and annual public reporting.
2. Standardise a primary care prevention and early detection package that links validated risk assessment to treatment, recall, and equity-sensitive outreach.
3. Implement explicit ACS, HF, and AF pathways with measurable hand-offs from emergency or primary care through specialist treatment, discharge, rehabilitation, and follow-up.
4. Make rehabilitation referral automatic for eligible patients and monitor enrolment and completion rather than referral alone.
5. Establish interoperable registries and a public dashboard using harmonised clinical, implementation, cost, data quality, and equity indicators.
6. Place digital and AI tools under lifecycle governance, including local validation, cybersecurity, reimbursement, human oversight, maintenance, and suspension criteria.
7. Test the integrated model through a staged Cyprus pilot with prespecified success, adaptation, failure, and transferability gates before wider adoption.

## 8. Limitations and Conclusions

**Limitations.** This is a structured critical narrative review, not a systematic review, meta-analysis, clinical guideline, or formal health technology assessment. Source types and the levels of certainty vary, and several Safe Hearts flagships have not yet undergone prospective evaluation. The ESC–AHA/ACC comparison is selective and implementation-focused. The Cyprus proposal is hypothesis-generating, and the suggested indicators and decision gates require stakeholder agreement, feasibility testing, validated definitions, and economic evaluation before adoption.

**Conclusions.** The Safe Hearts Plan can add value if it becomes an accountable delivery programme rather than another layer of recommendations. Europe should first standardise proven prevention, disease-specific treatment, rehabilitation, and registry reporting; introduce screening and AI only through governed pathways that measure net patient benefit, cost, and equity; and require national plans with funding, ownership, and a compact public scorecard. A staged Cyprus pilot can test the complete model, but expansion should occur only when prespecified clinical, implementation, economic, and equity thresholds are met and the receiving system’s financing, workforce, digital, rehabilitation, and governance conditions are explicit. Success should be judged by improved outcomes and reduced disparities—not endorsement, diagnostic yield, or technology adoption alone.

## Figures and Tables

**Table 1:** The evidence maturity and implementation readiness of the ten Safe Hearts flagship actions.

Safe Hearts Flagship Action	Predominant Evidentiary Basis	Readiness	Critical Implementation Test
1. ‘EU Cares for Your Heart’ communication initiative	Implementation and health communication evidence	Ready with evaluation	Does reach translate into risk assessment, treatment uptake, and equitable engagement rather than awareness alone?
2. Information on food processing	Public health and nutritional epidemiology; evolving intervention evidence	Ready with adaptation	Are labels understandable, behaviour-changing, and aligned with wider food environment policy?
3. Modernised tobacco legislation	Mature population prevention evidence	Ready for standardisation	Are exposure, cessation support, enforcement, and inequalities monitored together?
4. Tools and financial measures for primary prevention and food reformulation	Guideline-supported risk factor management plus policy evidence	Ready with adaptation	Do payment and procurement mechanisms improve blood pressure, lipid, diabetes, obesity, and smoking outcomes?
5. Council recommendation on respiratory vaccination	Clinical and guideline evidence in relevant risk groups [20]	Ready with adaptation	Can eligible cardiovascular populations be identified and vaccinated through routine pathways?
6. EU cardiovascular health check protocol	Risk estimation evidence; mixed screening and economic evidence	Pilot before scale	Does the protocol improve treated risk and patient outcomes without unacceptable overdiagnosis, downstream testing, or opportunity cost?
7. Council recommendation on personalised treatment and monitoring	Guideline-supported therapy; implementation-dependent monitoring	Ready with adaptation	Are treatment intensification, adherence, follow-up, and patient-reported outcomes delivered consistently?
8. Artificial intelligence and digital health incubator	Emerging technology and early implementation evidence	Governed pilots only	Are models externally validated, interoperable, secure, reimbursed, accepted, maintained, and outcome-improving?
9. Cardiovascular inequality dashboard	Implementation, registry, and health equity evidence	Ready if data are complete	Are indicators timely, stratified, publicly accountable, and linked to corrective action?
10. Cardiovascular research and innovation roadmap	Expert and policy consensus	Strategically ready	Are funds linked to prespecified clinical, implementation, economic, and equity deliverables?

Note: Categories are narrative implementation judgements and not formal grades of clinical recommendation. A single flagship can draw on more than one evidence type; this table identifies the dominant basis and the principal decision required for implementation.

**Table 2:** Staged roadmap and decision gates for Cyprus Safe Hearts pilot.

Phase	Indicative Period	Core Actions	Decision Gate
Design	0–6 months	Appoint accountable leadership; map services and inequalities; agree on disease-specific pathways, minimum dataset, financing, ethics, cybersecurity, and evaluation protocol.	Proceed only when governance, workforce, data access, funding, and baseline measures are documented.
Pilot delivery	7–24 months	Implement the prevention package and ACS, HF, and AF pathways in selected districts or provider networks; activate automatic rehabilitation referral, registry capture, and supported digital follow-up.	Continue only if reach, adoption, safety, fidelity, and data completeness meet prespecified thresholds without widening inequalities.
Comparative evaluation	24–36 months	Assess clinical outcomes, patient-reported outcomes, costs, workforce effects, unintended consequences, and variation against baseline or suitable comparators.	Adapt, discontinue, or prepare scale-up according to net benefit, affordability, and implementation findings—not diagnostic yield alone.
Conditional scale-up	>36 months	Extend successful components nationally; publish an implementation manual, negative findings, resource requirements, and boundary conditions for European adaptation.	Transfer a function only when receiving systems can reproduce its financing, workforce, digital, rehabilitation, and governance requirements.

Note: Timings are indicative and should be finalised with Cypriot stakeholders. Thresholds should be prespecified in the protocol rather than selected after pilot results are known.

**Table 3:** Disease-specific implementation crosswalk between selected ESC and AHA/ACC guidance.

Pathway	ESC Anchor	AHA/ACC Comparator	Minimum Delivery Bundle	Illustrative Pathway Indicator
Prevention and hypertension	2021 ESC prevention and 2019 ESC/EAS lipids; 2024 ESC: hypertension ≥ 140/90 mmHg, elevated BP 120–139/70–89 mmHg, and treated SBP target 120–129 mmHg if tolerated [12,13,14]	2019 ACC/AHA prevention; 2025 AHA/ACC: stage 1 hypertension 130–139 or 80–89 mmHg and general treatment goal < 130/80 mmHg [27,76]	Validated regional risk assessment; repeated BP confirmation; lipid, diabetes, renal, smoking, and obesity assessment; treatment, adherence support, and recall.	Eligible adults with documented risk assessment and controlled BP/LDL-C, stratified by equity variables.
Acute coronary syndrome (ACS)	2023 ESC ACS guideline [77]	2025 ACC/AHA multisociety ACS guideline [78]	EMS–emergency department–catheterisation coordination; rapid ECG and troponin pathways; reperfusion/invasive care; antithrombotic and lipid therapy; discharge bundle, rehabilitation, and early follow-up.	Time to reperfusion/invasive assessment; complete discharge therapy; rehabilitation completion; 30-day and 1-year events.
Heart failure (HF)	2021 ESC HF and 2023 focused update: SGLT2 inhibitors Class I, level A in HFmrEF/HFpEF [79,80]	2022 AHA/ACC/HFSA: SGLT2 inhibitors Class IIa in HFmrEF/HFpEF [81]	Access to natriuretic peptides and echocardiography; phenotype classification; rapid initiation and titration of indicated therapy; congestion and renal monitoring; post-discharge review; rehabilitation and advanced care planning.	Eligible patients receiving indicated therapy at tolerated doses; 7–14-day review; 30-day readmission; quality of life.
Atrial fibrillation (AF)	2024 ESC AF: CHA2DS2-VA; anticoagulation recommended at score ≥ 2 and considered at score 1 [16]	2023 ACC/AHA/ACCP/HRS: anticoagulation guided by estimated annual thromboembolic risk ≥ 2%, using a validated score such as CHA2DS2-VASc [82]	ECG confirmation; thromboembolic and bleeding risk review; anticoagulation when indicated; comorbidity and risk factor management; symptom-directed rate/rhythm control; recurrent reassessment.	Appropriate anticoagulation; time to confirmatory ECG; risk factor management; stroke, bleeding, and patient-reported symptoms.

Note: This table compares implementation functions and highlights selected material differences that alter diagnostic labels, treatment targets, or eligibility. It is not an exhaustive comparison of every class or level of recommendation; full clinical decisions should follow the current guideline applicable to the jurisdiction and patient.

**Table 4:** Proposed core key performance indicators for the Safe Hearts Plan.

Domain	Core Indicator	Minimum Reporting Specification	Why It Matters
Population outcome	Premature cardiovascular mortality	Age-standardised rate and change from baseline; national and subnational; stratified by sex and socioeconomic/geographic variables.	Tests whether delivery ultimately changes population harm.
Prevention reach	Risk factor assessment coverage	Eligible population with recent BP, lipids, glycaemia/diabetes risk, smoking status, renal risk, and documented validated risk estimate where applicable.	Identifies missed opportunities before disease appears.
Risk factor control	Control among treated/eligible populations	BP and LDL-C control; smoking cessation; diabetes and obesity management, using prespecified guideline-aligned definitions and contraindication exclusions.	Measures conversion of detection into effective management.
Disease-specific care	Guideline-directed therapy and pathway timeliness	ACS, HF, and AF bundles with eligible denominators, contraindications, timing, persistence, and major safety outcomes.	Avoids hiding disease-specific delivery inside aggregate CVD measures.
Rehabilitation	Referral, enrolment, and completion	All eligible patients; centre-based, home-based, or telerehabilitation; reasons for non-participation; equity stratification.	Distinguishes automatic referral from meaningful participation.
Clinical outcomes	Recurrent events and admissions	30-day and 1-year recurrent MI/stroke, HF readmission, major bleeding where relevant, and patient-reported health status.	Captures benefit and harm beyond process completion.
Data quality	Registry completeness and timeliness	Coverage of eligible cases, completeness of required fields, linkage success, validation error, and reporting lag.	Determines whether accountability measures are trustworthy.
Implementation and equity	Reach, adoption, fidelity, sustainability, and disparity	Provider and patient reach; pathway adherence; cost; continuation after initial funding; all core measures stratified by relevant population groups.	Shows whether an effective pathway is deliverable and fairly distributed.

Abbreviations: AF, atrial fibrillation; ACS, acute coronary syndrome; BP, blood pressure; CVD, cardiovascular disease; HF, heart failure; LDL-C, low-density lipoprotein cholesterol; MI, myocardial infarction.

## Data Availability

No new data were created or analysed in this study. Data sharing is not applicable to this article.

## Abbreviations

The following abbreviations are used in this manuscript:

ACC	American College of Cardiology
ACS	Acute coronary syndrome
AF	Atrial fibrillation
AHA	American Heart Association
AI	Artificial intelligence
BP	Blood pressure
CAC	Coronary artery calcium
CVD	Cardiovascular disease
ECG	Electrocardiogram
EHR	Electronic health record
EMS	Emergency medical services
ESC	European Society of Cardiology
EU	European Union
GESY	General Healthcare System
HF	Heart failure
HFmrEF	Heart failure with mildly reduced ejection fraction
HFpEF	Heart failure with preserved ejection fraction
KPI	Key performance indicator
LDL-C	Low-density lipoprotein cholesterol
MHI	Modified Haller index
MI	Myocardial infarction
QALY	Quality-adjusted life-year
SBP	Systolic blood pressure
SCORE2	Systematic Coronary Risk Evaluation 2
SCORE2-OP	Systematic Coronary Risk Evaluation 2-Older Persons
SGLT2	Sodium–glucose cotransporter 2

## References

[1] Timmis A., Vardas P., Townsend N., Torbica A., Katus H., De Smedt D., Gale C.P., Maggioni A.P., Petersen S.E., Huculeci R. (2022). European Society of Cardiology: Cardiovascular disease statistics 2021. Eur. Heart J..

[2] Townsend N., Kazakiewicz D., Wright F.L., Timmis A., Huculeci R., Torbica A., Gale C.P., Achenbach S., Weidinger F., Vardas P. (2022). Epidemiology of cardiovascular disease in Europe. Nat. Rev. Cardiol..

[3] Roth G.A., Mensah G.A., Johnson C.O., Addolorato G., Ammirati E., Baddour L.M., Barengo N.C., Beaton A.Z., Benjamin E.J., Benziger C.P. (2020). Global burden of cardiovascular diseases and risk factors, 1990–2019. J. Am. Coll. Cardiol..

[4] Vaduganathan M., Mensah G.A., Turco J.V., Fuster V., Roth G.A. (2022). The global burden of cardiovascular diseases and risk: A compass for future health. J. Am. Coll. Cardiol..

[5] World Health Organization Cardiovascular Diseases (CVDs): Fact Sheet. https://www.who.int/news-room/fact-sheets/detail/cardiovascular-diseases-(cvds).

[6] OECD (2025). The State of Cardiovascular Health in the European Union.

[7] Mannoh I., Hussien M., Commodore-Mensah Y., Michos E.D. (2021). Impact of social determinants of health on cardiovascular disease prevention. Curr. Opin. Cardiol..

[8] Marmot M. (2005). Social determinants of health inequalities. Lancet.

[9] WHO Commission on Social Determinants of Health (2008). Closing the Gap in a Generation: Health Equity Through Action on the Social Determinants of Health.

[10] Luengo-Fernandez R., Walli-Attaei M., Gray A., Torbica A., Maggioni A.P., Huculeci R., Bairami F., Aboyans V., Timmis A.D., Vardas P. (2023). Economic burden of cardiovascular diseases in the European Union: A population-based cost study. Eur. Heart J..

[11] Wilkins E., Wilson L., Wickramasinghe K., Bhatnagar P., Leal J., Luengo-Fernandez R., Burns R., Rayner M., Townsend N. (2017). European Cardiovascular Disease Statistics 2017.

[12] Visseren F.L.J., Mach F., Smulders Y.M., Carballo D., Koskinas K.C., Bäck M., Benetos A., Biffi A., Boavida J.M., Capodanno D. (2021). 2021 ESC Guidelines on cardiovascular disease prevention in clinical practice. Eur. Heart J..

[13] Mach F., Baigent C., Catapano A.L., Koskinas K.C., Casula M., Badimon L., Chapman M.J., De Backer G.G., Delgado V., Ference B.A. (2020). 2019 ESC/EAS Guidelines for the management of dyslipidaemias. Eur. Heart J..

[14] McEvoy J.W., McCarthy C.P., Bruno R.M., Brouwers S., Canavan M.D., Ceconi C., Christodorescu R.M., Clinthorne J., Cosentino F., Crousillat D.R. (2024). 2024 ESC Guidelines for the management of elevated blood pressure and hypertension. Eur. Heart J..

[15] Vrints C., Andreotti F., Koskinas K.C., Rossello X., Adamo M., Ainslie J., Banning A.P., Budaj A., Buechel R.R., Chiariello G.A. (2024). 2024 ESC Guidelines for the management of chronic coronary syndromes. Eur. Heart J..

[16] Van Gelder I.C., Rienstra M., Bunting K.V., Casado-Arroyo R., Caso V., Crijns H.J.G.M., De Potter T.J.R., Dwight J., Guasti L., Hanke T. (2024). 2024 ESC Guidelines for the management of atrial fibrillation. Eur. Heart J..

[17] European Commission (2025). Communication from the Commission to the European Parliament, the Council, the European Economic and Social Committee and the Committee of the Regions on an EU Cardiovascular Health Plan: The Safe Hearts Plan.

[18] European Alliance for Cardiovascular Health The European Safe Hearts Plan. https://www.cardiovascular-alliance.eu/.

[19] Georghiou G.P., Georghiou P., Georgiou A., Xanthopoulos A., Lambropoulos K., Triposkiadis F. (2025). The European Union Must Act Effectively for a New Plan of Action to Improve the Cardiovascular Health Burden of European citizens: A Narrative Review. J. Biomed. Res. Environ. Sci..

[20] Modin D., Lassen M.C.H., Claggett B., Johansen N.D., Keshtkar-Jahromi M., Skaarup K.G., Solomon S.D., Køber L., Schou M., Torp-Pedersen C. (2023). Influenza vaccination and cardiovascular events in patients with ischaemic heart disease and heart failure: A meta-analysis. Eur. J. Heart Fail..

[21] WHO Regional Office for Europe (2021). European Programme of Work, 2020–2025: United Action for Better Health.

[22] WHO Regional Office for Europe (2016). Action Plan for the Prevention and Control of Noncommunicable Diseases in the WHO European Region.

[23] European Commission (2022). Healthier Together—EU Non-Communicable Diseases Initiative.

[24] European Commission (2021). Europe’s Beating Cancer Plan.

[25] Yusuf S., Hawken S., Ounpuu S., Dans T., Avezum A., Lanas F., McQueen M., Budaj A., Pais P., Varigos J. (2004). Effect of potentially modifiable risk factors associated with myocardial infarction in 52 countries (the INTERHEART study): Case-control study. Lancet.

[26] Lloyd-Jones D.M., Allen N.B., Anderson C.A.M., Black T., Brewer L.C., Foraker R.E., Grandner M.A., Lavretsky H., Perak A.M., Sharma G. (2022). Life’s Essential 8: Updating and enhancing the American Heart Association’s construct of cardiovascular health. Circulation.

[27] Arnett D.K., Blumenthal R.S., Albert M.A., Buroker A.B., Goldberger Z.D., Hahn E.J., Himmelfarb C.D., Khera A., Lloyd-Jones D., McEvoy J.W. (2019). 2019 ACC/AHA Guideline on the Primary Prevention of Cardiovascular Disease. Circulation.

[28] Stone N.J., Smith S.C., Orringer C.E., Rigotti N.A., Navar A.M., Khan S.S., Jones D.W., Goldberg R., Mora S., Blaha M.J. (2022). Managing atherosclerotic cardiovascular risk in young adults: JACC State-of-the-Art Review. J. Am. Coll. Cardiol..

[29] Genovesi S., Giussani M., Orlando A., Battaglino M.G., Nava E., Parati G. (2019). Prevention of cardiovascular diseases in children and adolescents. High Blood Press. Cardiovasc. Prev..

[30] NCD Risk Factor Collaboration (2021). Worldwide trends in hypertension prevalence and progress in treatment and control from 1990 to 2019. Lancet.

[31] Mills K.T., Stefanescu A., He J. (2020). The global epidemiology of hypertension. Nat. Rev. Nephrol..

[32] Whelton P.K., Carey R.M., Aronow W.S., Casey D.E., Collins K.J., Dennison Himmelfarb C., DePalma S.M., Gidding S., Jamerson K.A., Jones D.W. (2018). 2017 ACC/AHA Guideline for the Prevention, Detection, Evaluation, and Management of High Blood Pressure in Adults. Hypertension.

[33] Ference B.A., Ginsberg H.N., Graham I., Ray K.K., Packard C.J., Bruckert E., Hegele R.A., Krauss R.M., Raal F.J., Schunkert H. (2017). Low-density lipoproteins cause atherosclerotic cardiovascular disease. 1. Evidence from genetic, epidemiologic, and clinical studies. A consensus statement from the European Atherosclerosis Society Consensus Panel. Eur. Heart J..

[34] Sabatine M.S., Giugliano R.P., Keech A.C., Honarpour N., Wiviott S.D., Murphy S.A., Kuder J.F., Wang H., Liu T., Wasserman S.M. (2017). Evolocumab and clinical outcomes in patients with cardiovascular disease. N. Engl. J. Med..

[35] Silverman M.G., Ference B.A., Im K., Wiviott S.D., Giugliano R.P., Grundy S.M., Braunwald E., Sabatine M.S. (2016). Association between lowering LDL-C and cardiovascular risk reduction among different therapeutic interventions: A systematic review and meta-analysis. JAMA.

[36] American Diabetes Association Professional Practice Committee (2025). Standards of Care in Diabetes—2025. Diabetes Care.

[37] GBD 2021 Diabetes Collaborators (2023). Global, regional, and national burden of diabetes from 1990 to 2021. Lancet.

[38] Cosentino F., Grant P.J., Aboyans V., Bailey C.J., Ceriello A., Delgado V., Federici M., Filippatos G., Grobbee D.E., Hansen T.B. (2020). 2019 ESC Guidelines on diabetes, pre-diabetes, and cardiovascular diseases developed in collaboration with the EASD. Eur. Heart J..

[39] González-Muniesa P., Martínez-González M.A., Hu F.B., Després J.P., Matsuzawa Y., Loos R.J.F., Moreno L.A., Bray G.A., Martinez J.A. (2017). Obesity. Nat. Rev. Dis. Primers.

[40] Bray G.A., Kim K.K., Wilding J.P.H., World Obesity Federation (2017). Obesity: A chronic relapsing progressive disease process. A position statement of the World Obesity Federation. Obes. Rev..

[41] Wilding J.P.H., Batterham R.L., Calanna S., Davies M., Van Gaal L.F., Lingvay I., McGowan B.M., Rosenstock J., Tran M.T.D., Wadden T.A. (2021). Once-weekly semaglutide in adults with overweight or obesity. N. Engl. J. Med..

[42] Anderson L., Thompson D.R., Oldridge N., Zwisler A.D., Rees K., Martin N., Taylor R.S. (2016). Exercise-based cardiac rehabilitation for coronary heart disease. Cochrane Database Syst. Rev..

[43] Taylor R.S., Dalal H.M., McDonagh S.T.J. (2022). The role of cardiac rehabilitation in improving cardiovascular outcomes. Nat. Rev. Cardiol..

[44] Ambrosetti M., Abreu A., Corrà U., Davos C.H., Hansen D., Frederix I., Iliou M.C., Pedretti R.F.E., Schmid J.P., Vigorito C. (2021). Secondary prevention through comprehensive cardiovascular rehabilitation: From knowledge to implementation. 2020 update. A position paper from the Secondary Prevention and Rehabilitation Section of the European Association of Preventive Cardiology. Eur. J. Prev. Cardiol..

[45] SCORE2 Working Group and ESC Cardiovascular Risk Collaboration (2021). SCORE2 risk prediction algorithms: New models to estimate 10-year risk of cardiovascular disease in Europe. Eur. Heart J..

[46] SCORE2-OP Working Group and ESC Cardiovascular Risk Collaboration (2021). SCORE2-OP risk prediction algorithms: Estimating incident cardiovascular event risk in older persons. Eur. Heart J..

[47] European Society of Cardiology SCORE2 and SCORE2-OP Cardiovascular Risk Estimation Charts. https://www.escardio.org/Guidelines/Clinical-Practice-Guidelines/CVD-Prevention-Guidelines.

[48] Svennberg E., Friberg L., Frykman V., Al-Khalili F., Engdahl J., Rosenqvist M. (2021). Clinical outcomes in systematic screening for atrial fibrillation (STROKESTOP): A multicentre, parallel group, unmasked, randomised controlled trial. Lancet.

[49] Svendsen J.H., Diederichsen S.Z., Højberg S., Krieger D.W., Graff C., Kronborg C., Olesen M.S., Nielsen J.B., Holst A.G., Brandes A. (2021). Implantable loop recorder detection of atrial fibrillation to prevent stroke (The LOOP Study): A randomised controlled trial. Lancet.

[50] Perez M.V., Mahaffey K.W., Hedlin H., Rumsfeld J.S., Garcia A., Ferris T., Balasubramanian V., Russo A.M., Rajmane A., Cheung L. (2019). Large-scale assessment of a smartwatch to identify atrial fibrillation. N. Engl. J. Med..

[51] Guo Y., Wang H., Zhang H., Liu T., Liang Z., Xia Y., Yan L., Xing Y., Shi H., Li S. (2019). Mobile photoplethysmographic technology to detect atrial fibrillation. J. Am. Coll. Cardiol..

[52] Scherrenberg M., Falter M., Abreu A., Aktaa S., Busnatu S., Casado-Arroyo R., Hansen D., Frederix I., De Sutter J., Kemps H. (2025). Standards for cardiac telerehabilitation. Eur. Heart J..

[53] Topol E.J. (2019). Deep Medicine: How Artificial Intelligence Can Make Healthcare Human Again.

[54] Cowie M.R., Bax J., Bruining N., Cleland J.G.F., Koehler F., Malik M., Pinto F., van der Velde E. (2016). e-Health: A position statement of the European Society of Cardiology. Eur. Heart J..

[55] World Health Organization (2021). Global Strategy on Digital Health 2020–2025.

[56] European Society of Cardiology (2024). ESC Digital Health Strategy.

[57] Topol E.J. (2019). High-performance medicine: The convergence of human and artificial intelligence. Nat. Med..

[58] Rajpurkar P., Chen E., Banerjee O., Topol E.J. (2022). AI in health and medicine. Nat. Med..

[59] Johnson K.W., Torres Soto J., Glicksberg B.S., Shameer K., Miotto R., Ali M., Ashley E., Dudley J.T. (2018). Artificial intelligence in cardiology. J. Am. Coll. Cardiol..

[60] Attia Z.I., Kapa S., Lopez-Jimenez F., McKie P.M., Ladewig D.J., Satam G., Pellikka P.A., Enriquez-Sarano M., Noseworthy P.A., Munger T.M. (2019). Screening for cardiac contractile dysfunction using an artificial intelligence-enabled electrocardiogram. Nat. Med..

[61] Batra G., Aktaa S., Wallentin L., Maggioni A.P., Wilkinson C., Casadei B., Gale C.P., Norrving B., Gouveia E., Huber K. (2023). Methodology for the development of international clinical data standards for common cardiovascular conditions: European Unified Registries for Heart Care Evaluation and Randomised Trials (EuroHeart). Eur. Heart J. Qual. Care Clin. Outcomes.

[62] European Society of Cardiology ESC Atlas of Cardiology. https://www.escardio.org/Research/ESC-Atlas-of-cardiology.

[63] Rajagopalan S., Al-Kindi S.G., Brook R.D. (2018). Air pollution and cardiovascular disease: JACC State-of-the-Art Review. J. Am. Coll. Cardiol..

[64] Newby D.E., Mannucci P.M., Tell G.S., Baccarelli A.A., Brook R.D., Donaldson K., Forastiere F., Franchini M., Franco O.H., Graham I. (2015). Expert position paper on air pollution and cardiovascular disease. Eur. Heart J..

[65] Romanello M., Di Napoli C., Drummond P., Green C., Kennard H., Lampard P., Scamman D., Arnell N., Ayeb-Karlsson S., Ford L.B. (2023). The 2023 report of the Lancet Countdown on health and climate change: The imperative for a health-centred response in a world facing irreversible harms. Lancet.

[66] Aitken W.W., Brown S.C., Comellas A.P. (2022). Climate change and cardiovascular health. J. Am. Heart Assoc..

[67] Chaseling G.K., Uchmanowicz I., Bäck M., Miró Ò., Tokmakova M., Ljungman P., Thiele H., Aboyans V., Boriani G., Casadei B. (2025). Heat extremes and cardiovascular diseases: A scientific statement of the Association of Cardiovascular Nursing & Allied Professions, Association for Acute Cardiovascular Care, European Association of Preventive Cardiology, Heart Failure Association, European Heart Rhythm Association of the ESC, the ESC Council on Hypertension, the ESC Council on Stroke, and the ESC Working Group on Cardiovascular Pharmacotherapy. Eur. Heart J..

[68] Charalampous P., Gorasso V., Plass D., Pires S.M., von der Lippe E., Naghavi M., Eikemo T.A., GBD 2017 Cyprus Collaborators (2021). Burden of non-communicable diseases in Cyprus, 1990–2017: Findings from the Global Burden of Disease 2017 study. Arch. Public Health.

[69] Kyprianidou M., Panagiotakos D.B., Makris K.C., Kambanaros M., Christophi C.A., Giannakou K. (2022). The lifestyle profile of individuals with cardiovascular and endocrine diseases in Cyprus: A hierarchical, classification analysis. Nutrients.

[70] OECD/European Observatory on Health Systems and Policies (2023). Cyprus: Country Health Profile 2023.

[71] Glasgow R.E., Harden S.M., Gaglio B., Rabin B., Smith M.L., Porter G.C., Ory M.G., Estabrooks P.A. (2019). RE-AIM planning and evaluation framework: Adapting to new science and practice with a 20-year review. Front. Public Health.

[72] Proctor E.K., Silmere H., Raghavan R., Hovmand P., Aarons G., Bunger A., Griffey R., Hensley M. (2011). Outcomes for implementation research: Conceptual distinctions, measurement challenges, and research agenda. Adm. Policy Ment. Health.

[73] Greenhalgh T., Robert G., Macfarlane F., Bate P., Kyriakidou O. (2004). Diffusion of innovations in service organizations: Systematic review and recommendations. Milbank Q..

[74] European Commission Horizon Europe: The EU Research and Innovation Programme, Health Cluster. https://research-and-innovation.ec.europa.eu/funding/funding-opportunities/funding-programmes-and-open-calls/horizon-europe_en.

[75] European Commission Digital Europe Programme. https://digital-strategy.ec.europa.eu/en/activities/digital-programme.

[76] Jones D.W., Ferdinand K.C., Taler S.J., Johnson H.M., Shimbo D., Abdalla M., Agarwal R., Albert N.M., Ballantyne C.M., Bello N.A. (2025). 2025 AHA/ACC/AANP/AAPA/ABC/ACCP/ACPM/AGS/AMA/ASPC/NMA/PCNA/SGIM Guideline for the Prevention, Detection, Evaluation, and Management of High Blood Pressure in Adults. J. Am. Coll. Cardiol..

[77] Byrne R.A., Rossello X., Coughlan J.J., Barbato E., Berry C., Chieffo A., Claeys M.J., Dan G.A., Dweck M.R., Galbraith M. (2023). 2023 ESC Guidelines for the management of acute coronary syndromes. Eur. Heart J..

[78] Rao S.V., O’Donoghue M.L., Ruel M., Rab T., Tamis-Holland J.E., Alexander J.H., Baber U., Baker H., Cohen M.G., Cruz-Ruiz M. (2025). 2025 ACC/AHA/ACEP/NAEMSP/SCAI Guideline for the Management of Patients with Acute Coronary Syndromes. J. Am. Coll. Cardiol..

[79] McDonagh T.A., Metra M., Adamo M., Gardner R.S., Baumbach A., Böhm M., Burri H., Butler J., Čelutkienė J., Chioncel O. (2021). 2021 ESC Guidelines for the diagnosis and treatment of acute and chronic heart failure. Eur. Heart J..

[80] McDonagh T.A., Metra M., Adamo M., Gardner R.S., Baumbach A., Böhm M., Burri H., Butler J., Čelutkienė J., Chioncel O. (2023). 2023 Focused Update of the 2021 ESC Guidelines for the diagnosis and treatment of acute and chronic heart failure. Eur. Heart J..

[81] Heidenreich P.A., Bozkurt B., Aguilar D., Allen L.A., Byun J.J., Colvin M.M., Deswal A., Drazner M.H., Dunlay S.M., Evers L.R. (2022). 2022 AHA/ACC/HFSA Guideline for the Management of Heart Failure. Circulation.

[82] Joglar J.A., Chung M.K., Armbruster A.L., Benjamin E.J., Chyou J.Y., Cronin E.M., Deswal A., Eckhardt L.L., Goldberger Z.D., Gopinathannair R. (2024). 2023 ACC/AHA/ACCP/HRS Guideline for the Diagnosis and Management of Atrial Fibrillation. Circulation.

[83] Lyth J., Svennberg E., Bernfort L., Aronsson M., Frykman V., Al-Khalili F., Rosenqvist M., Levin L.Å. (2023). Cost-effectiveness of population screening for atrial fibrillation: The STROKESTOP study. Eur. Heart J..

[84] Kronborg C., Kongstad L.P., Diederichsen S.Z., Højberg S., Haugan K.J., Graff C., Brandes A., Lydiksen N.V., Køber L., Krieger D. (2026). The cost-effectiveness of implantable loop recorder detection of atrial fibrillation to prevent stroke in persons at high risk: An economic evaluation alongside a multicentre randomised controlled trial in Denmark (The LOOP Study). Eur. J. Health Econ..

[85] Boriani G., Laroche C., Diemberger I., Fantecchi E., Popescu M.I., Rasmussen L.H., Sinagra G., Petrescu L., Tavazzi L., Maggioni A.P. (2015). Asymptomatic atrial fibrillation: Clinical correlates, management, and outcomes in the EORP-AF Pilot General Registry. Am. J. Med..

[86] Sonaglioni A., Grasso E., Nicolosi G.L., Lombardo M. (2024). Modified Haller Index is inversely associated with asymptomatic status in atrial fibrillation patients undergoing electrical cardioversion: A preliminary observation. Minerva Cardiol. Angiol..

[87] European Parliament and Council of the European Union (2024). Regulation (EU) 2024/1689 laying down harmo-nised rules on artificial intelligence (Artificial Intelligence Act). Off. J. Eur. Union.

[88] European Parliament and Council of the European Union (2025). Regulation (EU) 2025/327 on the European Health Data Space. Off. J. Eur. Union.

